# 

**Published:** 1888-08-18

**Authors:** 


					(PR1GE ONE PENNY.
Nor-ggJ vol. iv.-
-August 18th, 1888.
[Registered at the General Post Office
as a Newspaper.]
H
Per Annum, 6b. 6d.. post free.
Monthly Parts, 6d.; post free, 7*d.
Ditto per Ann., 7s. 6d. post free.
A Weekly Institutional Journal of
Sricttct, /Ifotbtrim, IRurstitg, attb U^ijtlatttljtopg.

				

